# Consumer Perception and Acceptability of Plant-Based Alternatives to Chicken

**DOI:** 10.3390/foods11152271

**Published:** 2022-07-29

**Authors:** Laurel Ettinger, Anika Falkeisen, Sophie Knowles, Mackenzie Gorman, Sophie Barker, Rachael Moss, Matthew B. McSweeney

**Affiliations:** School of Nutrition and Dietetics, Acadia University, Wolfville, NS B4P 2K5, Canada; laurel.ettinger@acadiau.ca (L.E.); 139757f@acadiau.ca (A.F.); 154074k@acadiau.ca (S.K.); 153760g@acadiau.ca (M.G.); 142275b@acadiau.ca (S.B.); 145961m@acadiau.ca (R.M.)

**Keywords:** sensory analysis, consumer acceptance, meat analogue, alternative protein, imitation meat

## Abstract

The prevalence of plant-based alternatives (PBAs) to meat in the marketplace has been increasing in recent years due to consumer demand. One of these plant-based products has aimed to mimic chicken products, specifically chicken nuggets. However, few sensory studies have been conducted on these products. The objective of this study is to evaluate the sensory properties, acceptability, and consumer perception of these PBAs. Participants (*n* = 105) were asked to evaluate five PBAs and a control (chicken nugget) using hedonic scales and a check-all-that-apply question. They also answered an open-ended comment question about PBAs. The participants separated the control from the PBAs in terms of their hedonic scores and sensory properties. They separated the PBAs based on their textural properties and if they had off-flavors. Participants disliked PBAs that were associated with an aftertaste, as well as beany, fibrous, and chewy attributes. The participants believed the PBAs currently on the market did not successfully mimic a chicken nugget and that improvement is needed, but they did believe PBAs are environmentally friendly.

## 1. Introduction

Animal products have been a staple of diets in Western cultures for decades [1]. Historically, meat consumption has been and remains a sign of wealth [1]. Further, meat has been recommended as an essential part of a regular diet [2,3]. To meet demands of the world’s growing population, which is estimated to reach nine billion by 2050 [4], meat production has had to increase dramatically. From 1998 to 2018 alone, meat consumption increased by fifty-eight percent [5]. The consequence of this increase is a significant strain on the environment, mostly due to the production of greenhouse gas (GHG) emissions [6]. Globally, agriculture and food systems are responsible for more than twenty-four percent of all greenhouse gas (GHG) emissions, of which meat and dairy contribute significantly [7,8]. The combined strain of a growing population, increased meat consumption, and increased GHGs places a severe burden on the planet’s natural resources and is contributing substantially to climate change [1,6,9]. In contrast, plant-based diets, which often include plant-based alternatives (PBAs), are suspected to use fewer natural resources and reduce the overall impact on the environment [1,10]. Although findings vary between study methodology and specific plant-based diets, in comparison to meat-based diets, estimates consistently show that consumption of plant-based diets can decrease GHG emissions [1,11]. Given the concern for environmental sustainability along with individual preferences, consumers are changing their diet patterns to include more plant-based sources [12]. Research into identifying types and formulations of PBAs that will increase the transition of individuals’ diets from meat to plant-based is essential to maintaining the planet for future generations.

The current demand for PBAs has established a significant market for many food manufacturers [5]. The COVID-19 pandemic has also altered consumers’ diets and has led to a higher consumption of PBAs [13,14]. Commercial PBAs are available with a variety of protein sources, most commonly soy, wheat, legumes, and fungi, and are frequently combined [15,16]. These proteins are most often available in PBAs that are purely plant-based or combined with meat protein [9]. In an effort to appeal to more consumers, specifically meat eaters, food manufacturers are attempting to develop PBAs that better mimic meat [5]. Despite the abundance of PBAs available on the market, uptake from consumers remains poor [17].

In general, the acceptance of available PBAs is low compared to meat-based products [12] and a past study found that US consumers prefer a beef burger over a plant-based and blended burger with beef and mushrooms [9]. Of the many factors impacting consumer acceptance of PBAs, including cultural preference, perceived nutritional impact, familiarity, and processing and food neophobia [18], a significant factor is the sensory characteristics of these products [17]. A trend identified in this field indicates that consumers have prl-existing negative perceptions of the sensory aspects of PBAs [19]. Structurally, it is challenging to replicate the “meat-like” texture in a nonmeat product [20] and PBAs can be porous and loose [21]. Plant-based alternatives generally taste and look different than meat-based products, often leading to the addition of stabilizers, thickeners, dyes, and salt to achieve a meat-like structure [20]. Cordelle and colleagues [17] found that taste and appearance were the main drivers of persuasion to consume PBAs over meat-based products. The odor, taste, and texture of the PBAs had a marked impact on liking [17]. Overwhelmingly, the success of PBAs, or lack thereof, is dependent on the ability to create a meat-like experience [19,22]. Further sensory research is needed to identify the sensory properties that contribute to overall liking and acceptance of PBAs [6]. Specifically, more research needs to be conducted on PBAs created to mimic chicken, as many studies have focused on alternatives to beef.

Hedonic scales, specifically nine-point hedonic scales, are used to determine how much consumers like food items [23]. Furthermore, hedonic scales can be paired with a check-all-that-apply (CATA) question to evaluate which sensory properties are important to consumers, as well as which sensory properties are detracting from liking [24]. CATA presents the participants in the sensory trial with a list of sensory properties and the participants are asked to select which sensory properties apply to the sample [25]. CATA is a method that is easy for untrained consumers to understand and has led to reproductible results [25]. Additionally, CATA has been used to evaluate a variety of different plant-based products [26,27,28,29,30].

As such, the aim of this study is to explore Canadians’ sensory perception and acceptability of PBAs created to mimic chicken. Participants were asked to evaluate the PBAs using hedonic scales and a CATA question. In addition, the participants were asked their thoughts about PBAs using an open-ended comment question.

## 2. Materials and Methods

The study was reviewed and approved by the Acadia University Research Ethics Board (#13-72; 6 May 2021).

### 2.1. Samples

The samples were purchased from local grocery stores a week prior to the sensory trial. The samples were stored in a freezer (−18 °C) until the day of the sensory trial. All of the samples were labeled as plant-based chicken or plant-based alternatives to chicken nuggets (PBAs). The samples were included based on three different visits to grocery stores in the Annapolis Valley, Nova Scotia, Canada over three months. At each visit, the PBAs were recorded and those that were regularly available were selected to be included in the study. In addition, a chicken nugget sample (referred to as control in Table 1) was included in the trial. The ingredients of each sample are listed in Table 1 and the samples ranged in weight from 17 g to 25 g. Each sample was cooked in the oven following the directions on their packaging. The samples were removed from the oven and cooled to an internal temperature of 45 °C. Once cooled, whole samples were placed in transparent plastic cups and labeled with random three-digit codes. Samples were presented one at a time to the participants following a completely randomized design. Participants also received a glass of distilled water to cleanse their palates. The participants were seated in individual sensory booths and completed the testing using the Compusense^®^ Cloud software (Guelph, Ontario, Canada) and computers.

### 2.2. Participants

Participants were recruited in the Annapolis Valley Region of Nova Scotia, Canada via posted advertisements, word of mouth, and social media posts. Participants were screened for allergies and interest in consuming PBAs. In total, 105 participants were recruited for the study. Table 2 outlines the demographic details of the participants.

### 2.3. Sensory Procedure

The procedure for the sensory trial was adapted from Gorman and colleagues [31]. This study took place during the COVID-19 pandemic; therefore, participants were asked to self-identify if they had potential exposure to the virus or loss of smell and taste to ensure that health risks were minimized. Each participant completed an informed consent form. The participants were then presented with each blinded sample one at a time and asked to evaluate their liking of appearance, flavor, texture, and overall liking on a nine-point hedonic scale (1 = Dislike Extremely, 5 = Neither Like nor Dislike, and 9 = Like Extremely). The participants then answered a CATA question. The CATA question included the terms: sweet, salty, bitter, sour, savory, crunchy, hard, soft, juicy, crispy, beany, fatty, moist, wheaty, cardboard, dry, off-flavor, chicken, aftertaste, no aftertaste, rubbery, nutty, chewy, gummy, meaty, and fibrous. The attributes were included based on a literature review [6,16,27,29,32,33,34]. The attributes were randomized [25]. Participants also had the option to provide additional comments about the samples using an open-ended comment question. Once the participants finished evaluating the samples, they were asked an open-ended comment question, “What are your thoughts about plant-based alternatives to chicken?”. Participants were also asked questions regarding consumption of PBAs and chicken, as well as demographic questions.

### 2.4. Texture Profile Analysis

The texture of the cooked breaded samples was evaluated using a Texture Analyzer (TA.XT plus C Stable Micro Systems, Surrey, UK). The method described was adapted from Yuliarti and colleagues [34]. A cylindrical probe of 45 mm in diameter was used to conduct a 2-bite test. The sample was cut into a square with dimensions of 5 cm × 3 cm × 2 cm (length × width × height). The samples were compressed twice to 40% of their original height at a speed of 5.0 mm/s. The texture profile analysis was completed in triplicate.

### 2.5. Statistical Analysis

The results of the hedonic scores and CATA question were analyzed following the procedure outlined in Hayward and McSweeney [35]. Hedonic scores were evaluated using a 2-way random factor ANOVA, followed by a Tukey’s HSD test. A Cochran’s Q test and correspondence analysis were completed based on the results of the CATA question. A penalty lift analysis combined the results of the CATA question and overall liking scores. The responses to the open-ended comment questions were analyzed by highlighting important statements and identifying recurring themes. Participants’ beliefs were identified and then categorized in groups to reduce the number of concepts. The results were discussed among the authors to reach a consensus. The means and standard deviations of the results of the texture profile analysis were calculated. The results were then subjected to ANOVA, followed by a Tukey’s HSD test. All analyses were completed using XLSTAT software (New York, NY, USA) in Microsoft Excel™.

## 3. Results and Discussion

The mean hedonic scores are listed in Table 3. Overall, the PBAs were found to be significantly different in terms of their flavor, texture, and overall liking than the control (chicken) (*p* < 0.05). There was no significant difference in the participants’ liking of the appearance of the samples (*p* < 0.05). PBA1 and PBA5 were liked significantly more than PBA4 in terms of texture and overall liking (*p* < 0.05). PBA1′s main ingredient was wheat flour (Table 1), which was not significantly different from the other PBA made from wheat flour (PBA2). Wheat is one of the most prevalent ingredients used to produce meat analogs [36]. Participants were found to prefer the wheat flour PBAs over the PBA4 made from mainly textured soy protein. However, they did not dislike all soy-based PBAs, as PBA5 was made from soy protein isolate and liked by participants. Soy protein is a common ingredient in plant-based alternatives [37]. Unfortunately, as these are commercially available products, the processing method is unknown but many different techniques can be used to produce texturized soy protein [38]. The main ingredient of PBA3 was pea protein concentrate and it was not significantly different from the other PBAs in terms of texture or overall liking, but it did score the lowest for the participants’ liking of flavor. Pea protein has been associated with bitterness, beany, earthy, and astringency [39,40], which may have led to the participants’ disliking of the flavor of PBA3.

**Figure 1:**
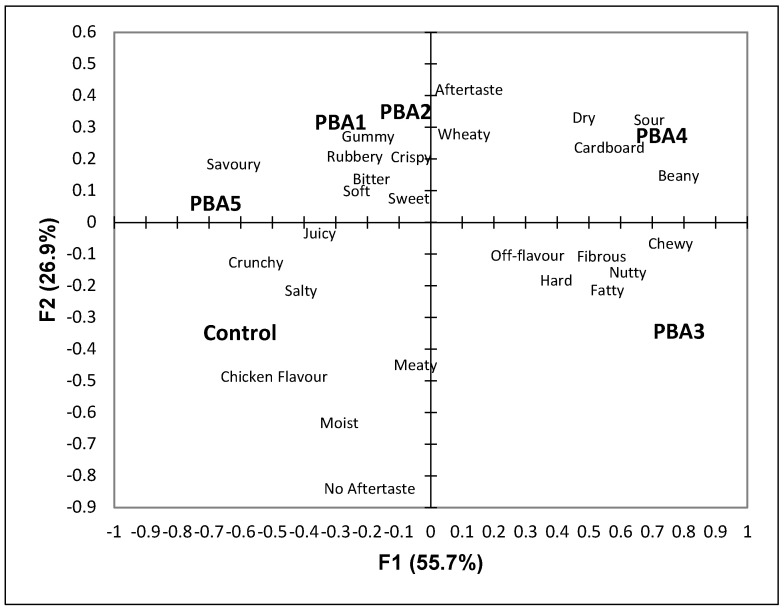
Biplot representation of the samples and sensory attributes on the first two dimensions of the correspondence analysis.

To investigate what attributes the participants associate with PBAs, a CATA question was used. The results of the CATA question were analyzed with a correspondence analysis and the first two dimensions can be found in Figure 1 (55.7% on the first dimension and 26.9% on the second dimension). The first dimension separated the sample based on the textural attributes chewy and fibrous on the positive side and crunchy on the negative side of the dimension. In a recent study on plant-based and chicken sausages, it was found that the plant-based ingredients influenced the textural properties [41]. Furthermore, textural attributes are highly correlated to consumer liking of chicken nuggets [42]. This result agrees with this study as the participants differentiated the samples based on textural properties. In addition, the sour and nutty attributes were on the positive side, while salty, chicken flavor, and savory were on the negative side. Salty taste is well-liked in chicken products [43], while sour and nutty are associated with plant-based proteins [44,45,46].

The second dimension separated the samples based on the presence of aftertaste. Plant-based proteins have been found to contribute an aftertaste to products [6], including pea protein [47] and soy protein [48]. The results of the correspondence analysis reinforce the results of the hedonic scores (Table 3), as the control was separated from the PBAs. Additionally, PBA1 and PBA2, which are both made from wheat flour, were grouped together. The soy PBAs (PBA4 and PBA5) were separated by the first dimension. PBA5 was associated with savory, crunchy, and juicy, while PBA4 was associated with sour, dry, and cardboard. PBA3, containing pea protein concentrate, was separated from all other samples and was associated with fatty, nutty, off-flavor, fibrous, and chewy. Past studies have found that pea protein can lead to off-flavors [39] and chewiness in meat analogs [34].

A penalty lift analysis was conducted to identify which sensory attributes impacted liking and disliking. Attributes such as crunchy, juicy, salty, crispy, no aftertaste, soft, and savory all positively impacted the participants’ overall liking scores. These attributes were all associated with the control, PBA5, and PBA1, which were also the most liked samples in the study. The results of the penalty lift analysis reinforce the importance of the textural attributes in consumers’ perception of chicken nuggets. In addition, crispness and crunchiness are important to consumers’ acceptance of foods such as chicken nuggets [49]. Crispness is a property that is well-liked by consumers in chicken nuggets [50] and, as stated above, saltiness is also well-liked [43]. Juiciness is another attribute that has been established as being important to consumer acceptance of chicken nuggets [51]. In the study by El-Anany and colleagues [51], as increasing amounts of chicken were substituted for cauliflower, the juiciness of the samples decreased as did the overall liking. Plant-based products have been found to be dense and chewy [52], and this could have led to soft (opposite of dense) increasing consumer liking. Furthermore, the responses to the open-ended comment question (Table 4) identified that dense and chewy are not well-liked by consumers.

**Figure 2:**
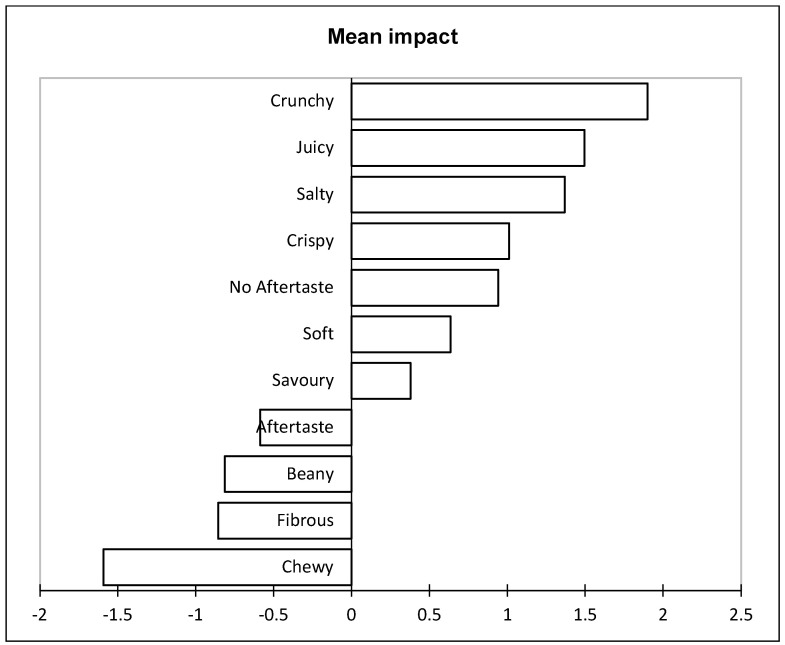
Penalty lift analysis of the sensory attributes and overall liking based on the participants’ evaluation of the samples.

The penalty lift analysis also indicated the importance of aftertaste to consumers, as the presence of aftertaste leads to consumer disliking (Figure 2) and no aftertaste leads to liking. As stated above, plant proteins can contribute an aftertaste, and this is not well-liked by consumers (Table 4). Beany, fibrous, and chewy also leads to the participants’ disliking the samples. Beany is a well-established flavor that is associated with soy protein [53], which agrees with this study, while beany was associated with PBA4, a soy-based PBA. Furthermore, the beany flavor is not well-liked by consumers in the Western world [54] and this was reinforced by the results of this study. Lastly, the fibrous attribute led to disliking. This result disagrees with a past study as researchers stated that the fibrous nature of plant-based protein leads to acceptable sensory properties [21]. The consumer dislike of the fibrous attribute may be due to the explanation given in the open-ended comment questions that the PBAs “fall apart” or “the texture is very off-putting”. They also could have selected fibrous as they did not feel the PBAs were able to mimic the textural attributes of chicken nuggets. Overall, properties associated with the control (chicken nuggets) led to consumer liking, while those that were linked with the PBAs were disliked.

After evaluating all of the samples, the participants were asked their thoughts about PBAs (Table 4). The majority of responses referred to the sensory properties of the PBAs (off-flavor, bland, different texture, and appearance). This may be because the participants had just completed a sensory trial about the PBAs. The participants indicated that PBAs did not taste like chicken and had many off-flavors. Meat analogs have been found to possess flavors that are different than chicken and meat-based products [37,39]. The participants specifically indicated that they do not like the taste of soy and repeatedly listed beany as an off-flavor. Furthermore, many participants stated that PBAs are bland and need more salt. This agrees with the results of the CATA question as salty was associated with the control sample. Participants did seem to rely on the CATA question to answer the open-ended comment question and listed many of the attributes that were included in the CATA question. This may be a limitation of this work and future studies may want to ask participants’ overall thoughts about a product category before having them evaluate the samples.

In contrast to the results of the sensory trial (Table 3), the participants indicated that they did not like the appearance of the PBAs in the open-ended comment question. Open-ended comment questions in the past have provided insight into the consumer perception [55] and, in this case, were able to identify that the appearance of PBAs was not well-liked. Open-ended comment questions are not restrictive and they allow the participants to answer them in their own style [56]. The comment question was able to identify an area of concern for the participants that would have not been identified if only hedonic scales were used. The participants did not like the appearance and discussed issues both with the exterior (“grains present in the coating”) and the interior (“inside looks gummy”). Product developers need to consider the effect of appearance on consumer liking. In addition, many participants identified that improvements are needed in the PBAs and stated that they are nothing like chicken. The participants in this study were looking for PBAs that mimic chicken and did not like flavors and textures that did not meet these criteria (see Figure 2). Meat analogs are meant to mimic the meat product they are based on (in this study, chicken nuggets) [16], and if they do not, they will not be well accepted by consumers.

In addition, in agreement with the results of the CATA question, the participants did not like the textural properties of the PBAs, and many stated that they have unacceptable textures (Table 4). Textural properties directly influence consumer liking of food products [57] and chicken nuggets [42]. The results identified by the texture analyzer also found differences in the texture (Table 5). PBA3 and PBA4 were significantly harder than the control (*p* < 0.05) and were liked significantly less than the control (Table 3). PBA5 was not as hard as the control (*p* < 0.05) and was not significantly different in terms of hedonic scores (*p* < 0.05). The chewiness scores follow a similar trend as the control was not significantly different than PBA1, but PBA2, PBA3, and PBA4 were all significantly chewier than the control (*p* < 0.05). These results also agree with the result of the CATA question as PBA2, PBA3, and PBA4 were on the positive side of the first dimension and associated with hard and chewy (Figure 1). This result also agrees with a study by Kamani and colleagues [41] that found that the partial and total substitution of chicken with plant protein in sausage affected the textural properties. Based on the results of the hedonic scales, CATA question, open-ended comment question, and texture analyzer, textural attributes are important to PBAs and participants currently perceive them to have different textures from their animal protein counterparts.

Lastly, the open-ended comment question identified that participants believe that PBAs are environmentally friendly and stated that it is “better than eating chicken in terms of sustainability”. The sustainability of plant-based proteins is well established [58,59,60]. Plant-based diets in comparison to those rich in animal proteins are more sustainable and use fewer natural resources [1]. Additionally, as stated by the participants, consumption of plant proteins can avoid concerns about the unethical treatment of animals [61]. This result also indicates that consumers are concerned with the sustainability of their food and this agrees with past studies [62,63,64].

This study compared the sensory properties of PBAs currently sold in Canada to a chicken-based control sample (chicken nuggets). Generally, the control was liked more than the PBAs by the participants and was associated with salty, chicken flavor, crunchy, and moist. The PBAs were separated from the control and the participants stated that the PBAs do not effectively mimic chicken in terms of textures or flavors. However, they did believe that PBAs are environmentally friendly. This study evaluated five PBAs mainly composed of wheat, soy, and pea protein, but there are many more PBAs made from different sources of plant protein that need to be evaluated for their sensory properties. Furthermore, this study asked consumers to evaluate chicken, and as such, those who avoid meat in their diet were not included. Future studies should ask consumers who do not eat meat to evaluate PBAs. As this study only included Canadian consumers in a specific region, future studies should investigate how consumers from different regions and countries evaluate PBAs. In addition, future studies should also investigate the chemical composition of PBAs, especially the moisture content, as well as their sensory properties.

## 4. Conclusions

The PBAs were found to be different from a chicken product and were separated based on their textural attributes, as well as their flavor and presence of aftertaste. The participants liked the PBAs that effectively mimicked chicken products and disliked those with off-flavors. Participants did not like the fibrous and beany attributes associated with the PBAs, and other PBAs that do not contain soy need to be investigated. Furthermore, participants identified that, currently, PBAs are not acceptable and identified that improvements are needed in the texture, flavor, and appearance. They also identified that PBAs are environmentally friendly and that they eat PBAs mainly because they are sustainable. Future studies should investigate the aftertaste associated with PBAs, as it was identified as the main contributor to participant disliking of PBAs. Overall, these results should help those working on the development of PBAs and identify the current consumer perception of plant-based alternatives to chicken.

## Figures and Tables

**Table 1:** Ingredients of the plant-based alternatives to chicken.

Sample	Ingredients
**Control**	*Chicken, water, toasted wheat crumbs, canola oil, chicken skin, modified corn starch, enriched wheat flour, corn flour, salt, soy protein isolate, wheat gluten, baking powder, guar gum, garlic powder, spice extract, and spices.*
**PBA1**	*Water, wheat flour, isolated soy protein, canola oil, wheat gluten, rice flour, oat bran, oats, methylcellulose, kamut flour, yeast extract, salt, dipotassium phosphate, potato starch, sea salt, natural flavors, sugar, cane sugar, amaranth flour, millet flour, quinoa flour, maltodextrin, soy sauce (water, soybeans, salt), color, white vinegar, spices, baking powder, dextrin, gum arabic, onion powder, garlic powder, soybean oil, yeast, pea protein, carrot powder, sugar beet fiber, magnesium oxide, ferric orthophosphate, niacinamide, zinc oxide, vitamin b12, calcium pantothenate, pyridoxine hydrochloride, thiamine hydrochloride, riboflavin.*
**PBA2**	*Water, wheat flour, soy protein isolate, vegetable oil, yellow corn flour, texturized wheat protein, modified corn starch, wheat protein isolate, potassium chloride, zinc oxide, ferric sulphate, niacinamide, cyanocobalamin, thiamine mononitrate, pyridoxine hydrochloride, pantothenic acid, maltodextrin, sugar dextrose, yeast extract, modified potato starch, corn starch, sunflower oil, salt, onion powder, garlic powder, tapioca dextrin, rice flour, baking powder, potato starch, paprika, L-lysine monohydrochloride, natural flavor, spices, sea salt, gum arabic, silicon dioxide.*
**PBA3**	*Water, pea protein concentrate, canola and coconut oil, toasted wheat crumbs, soy protein concentrate, modified cellulose, enriched wheat flour, modified corn starch, corn flour, brown rice protein concentrate, natural flavor, panko crumb (rice flour, pea protein concentrate, dextrose, baking soda), grain blend (wheat, oats, triticale, barley, rye), garlic powder, onion powder, soy protein isolate, yeast extract, corn starch, wheat gluten, salt, pea hull fiber, spices, rice starch, sea salt, sugar, tryptophan, rice flour, sodium phosphate, baking soda, guar gum, methionine, vitamins and minerals (calcium d-pantothenate, thiamine hydrochloride, riboflavin, niacinamide, pyridoxine hydrochloride (vitamin b6), cyanocobalamin (vitamin b12), folic acid, copper gluconate, ferric orthophosphate (iron), magnesium oxide, zinc oxide, potassium chloride).*
**PBA4**	*Water, textured soy protein, canola oil, textured wheat protein, wheat gluten, natural flavors, modified cellulose, soy protein, spices, yeast extract, salt, vitamin and mineral blend, toasted whole wheat crumbs, unbleached wheat flour, corn starch, corn flour, sea salt, baking powder, guar gum.*
**PBA5**	*Water, soy protein isolate, sunflower oil, wheat gluten, natural flavors, modified cellulose, organic cane sugar, yeast extract, onion powder, salt, garlic powder, pea protein isolate, enriched wheat flour, potato starch, organic cane sugar, yellow corn flour, yeast, salt, paprika, cream of tartar, baking soda.*

**Table 2:** Demographic details for the sensory trial (*n* = 105).

Characteristics	Sample Population (%)
*Age*	
19–20	7
21–29	25
30–39	24
40–49	19
50–59	15
60–65	10
*Gender*	
Male	40
Female	59
Prefer not to say	1
*Frequency of plant-based alternative to chicken consumption*	
Several times a week	3
At least once a week	11
Once a month	11
A few times a year	43
Never	32
*Frequency of chicken consumption*	
Several times a week	37
At least once a week	29
Once a month	11
A few times a year	10
Never	13

**Table 3:** Consumer mean liking scores (±standard deviation) for appearance, flavor, texture, and overall liking of the different samples.

Sample	Appearance	Flavor	Texture	Overall Liking
Control	5.8a ^1,2,3^ ± 1.1	7.1 a ± 1.0	6.9 a ± 1.0	7.0 a ± 1.2
PBA1	5.8 a ± 1.2	6.2 b ± 1.1	5.7 b ± 1.1	5.5 b ± 1.0
PBA2	5.4 a ± 1.0	5.8 b ± 1.0	5.3 bc ± 0.7	5.0 bc ± 0.9
PBA3	5.8 a ± 1.1	4.7 c ± 1.3	4.8 bc ± 0.8	4.9 bc ± 0.8
PBA4	5.7 a ± 0.9	5.2 bc ± 0.9	4.5 c ± 1.2	4.6 c ± 1.1
PBA5	5.6 a ± 1.0	5.7 b ± 1.0	5.7 b ± 1.1	5.7 b ± 1.0

^1^ Data input on a 9-point hedonic scale where 1 = Dislike Extremely and 9 = Like Extremely. ^2^ Means in the same column with the same letter (within the same trial) are not significantly different (*p* < 0.05). ^3^ *n* = 100.

**Table 4:** Main concepts identified about plant-based alternatives to chicken from the open-ended comment question asked after the sensory trial (Question: What are your thoughts about plant-based alternatives to chicken?).

Concept	Example of Responses
Off-flavor	Very strong flavour but not like chicken, grainy taste, strong aftertaste, they all have an aftertaste, beany, off-flavours, taste like soy-not good, sour, do not taste like chicken
Bland	Bland, not enough salt, has no taste at all, like cardboard, need dipping sauces for flavour, boring taste
Different texture	Dry, falls apart, coating never stays on, plasticky, dense, stick to your teeth, bad texture, the texture is very off-putting, feels like it is disintegrating in my mouth, very crumbly, gritty
Environmentally friendly	Good for the environment, better than eating chicken in terms of sustainability, reduces emissions, no animal abuse
Appearance	Never looks like chicken, weird appearance, some have grains present in the coating, look too uniform, do not look appealing, inside looks gummy
Improvements needed	Are nothing like chicken, more work needs to be done to make them taste like chicken, are not a substitute for chicken, improvements are needed before I will get them

**Table 5:** Textural properties of the cooked plant-based alternatives to chicken and the control.

Sample		Hardness (g)	Chewiness
Control	Mean SD	4914.3a ^1,2^ 400.9	2523.0 a 300.8
PBA1	Mean SD	4895.6 a 272.7	2783.1 ab 302.1
PBA2	Mean SD	5123.7 b 510.1	3860.2 ab 521.9
PBA3	Mean SD	5689.1 c 453.1	3142.1 b 430.1
PBA4	Mean SD	5590.1 c 432.1	3056.2 b 458.1
PBA5	Mean SD	3694.4 b 240.0	1112.5 c 164.6

^1^ Measurement was conducted in triplicate. ^2^ Means in the same column with the same letter (within the same trial) are not significantly different (*p* < 0.05).

## Data Availability

The data presented in this study are available on request from the corresponding author [M.M].
